# Evaluation of patient-reported outcomes in coronary CT angiography versus invasive coronary angiography

**DOI:** 10.1016/j.ejro.2026.100743

**Published:** 2026-03-24

**Authors:** Marc S. von der Stück, Robert Siepmann, Eric Corban, Michael Frick, Christiane Kuhl, Timm Dirrichs

**Affiliations:** a Department of Diagnostic and Interventional Radiology, University Hospital Aachen, Aachen, Germany; b Department of Cardiology, Pulmonology and Angiology, University Hospital Aachen, Aachen, Germany

**Keywords:** Computed tomography angiography, Coronary artery disease, Patient reported outcome measures, Coronary angiography

## Abstract

**Objectives:**

Coronary computed tomography angiography (CCTA) is the preferred non-invasive method for excluding coronary artery disease (CAD) in patients with an intermediate pre-test probability or stable CAD and often replaces invasive coronary angiography (ICA). However, patient acceptance and emotional responses to CCTA remain insufficiently studied. This study evaluated patient acceptance and emotional experiences during CCTA through patient-reported outcomes (PROs).

**Materials and methods:**

A total of 151 patients with a clinical indication for CCTA underwent standardized Dual-Source CT or Photon-Counting CT of the heart; 69 of these patients had previously undergone ICA. After the examination, a voluntary and anonymous PRO survey was completed using a standardized questionnaire comprising 27 items assessing emotions before and during the procedures. Responses were rated on 5-point Likert scales and visual analogue scales and compared with reported emotional responses to ICA and other semi- or non-invasive cardiac diagnostic procedures, including transesophageal echocardiography (TEE) and electrocardiography (ECG).

**Results:**

Overall, 82% (124/151) of patients rated CCTA as “very pleasant” or “pleasant”, compared to 29% (20/69) for ICA. 18% (27/151) rated CCTA as “very unpleasant” or “unpleasant”, versus 71% (49/69) for ICA. Mean examination comfort was significantly higher for CCTA (4.3/5) than for ICA (2.0/5; p < 0.001). Fear prior to examination was reported in 39.7% (60/151) for CCTA vs. 66.7% (46/69) for ICA (p < 0.001). Other non-invasive cardiac procedures were rated as mostly neutral compared to CCTA (range 2.2–3.3; 3 = neutral).

**Conclusion:**

CCTA is well tolerated and demonstrates significantly higher patient acceptance, with lower fear and discomfort, compared with ICA.

**Clinical relevance statement:**

This study underscores the clinical advantages of non-invasive CCTA beyond its established diagnostic value, highlighting its benefits with respect to patient comfort and compliance.

## Highlights

• Coronary CT angiography, as a procedure to rule out coronary artery disease, is well accepted by patients.
• Patient comfort is significantly higher for coronary CT angiography than invasive coronary angiography.
• Fear of coronary CT angiography is slight and reduces during the examination.

## 1 Introduction

Cardiac computed tomography, including coronary CT angiography (CCTA), has been endorsed by national medical societies and recommended in clinical practice guidelines for patients with intermediate risk of coronary artery disease (CAD) and for follow-up in stable CAD [1], [2], [3]. This shift has led to broader use of CCTA and a reduction in the need for diagnostic invasive coronary angiography (ICA) in many routine scenarios.

Beyond the exclusion of CAD, numerous other indications for CCTA exist, such as planning for transcatheter aortic valve implantation (TAVI). Numerous studies have compared the diagnostic performance of CCTA and ICA, as well as their complications and respective medical advantages and disadvantages [1], [2], [3], [4]. These studies have demonstrated that CCTA, for individuals with low to intermediate risk of obstructive CAD, matches ICA in terms of diagnostic sensitivity and specificity, yet with significantly fewer severe complications [1], [4].

However, patient acceptance of CCTA, especially in comparison to ICA, has received considerably less attention. Beyond clinical efficacy, patient comfort and experience are pivotal in healthcare, particularly in diagnostic work-up settings where patients may have a low pre-test probability of disease, limited symptoms, and where acceptance and adherence can influence completion of testing and downstream management. While anxiety during CCTA examination does not seem to significantly reduce image quality [5], a relaxed and comfortable environment can alleviate anxiety and stress during radiological imaging, improving patient compliance and imaging quality [6], [7], [8]. More comfortable diagnostic procedures may support adherence to recommended testing pathways and follow-up examinations.

Assessing patients´ acceptance of medical procedures is inherently challenging due to its subjective nature. It encompasses various factors, including perceived comfort, anxiety levels, and personal preferences, which are not easily quantifiable. Nevertheless, understanding and prioritizing patient perspectives are crucial for optimizing diagnostic strategies and ensuring patient-centered care. To this end, patient-reported outcomes (PROs) are utilized. PROs, respectively, their measurements with specific tools, are a well-accepted instrument to provide important information about patients’ subjective well-being, emotions, and acceptance of therapies or examinations [9], [10]. PRO focuses on the patient as the center of healthcare research and efforts [11], emphasizing the importance of involving patients in rating current and possibly improving future diagnostics and therapies.

Therefore, in the context of this study, we have focused on how patients perceive CCTA and how it compares to other diagnostic modalities, especially ICA. Furthermore, we aimed to find out potential discomforts associated with CCTA, intending to make the examination more comfortable for patients. To evaluate the acceptance of CCTA, patients who had undergone CCTA were asked to complete an anonymous questionnaire regarding their emotions, anxieties, concerns and inconveniencies before, during and after the examination. Medical complications and adverse events, such as contrast media side effects, were reported separately.

Some items were questioned in comparison between CCTA and ICA, as well as other semi- and non-invasive cardiologic diagnostic procedures, if patients had already undergone such in the past.

## 2 Materials & methods

### 2.1 Study design and cohort

This prospective clinical study in adult patients (> 18 years) was conducted between March 2022 and May 2025 at a tertiary care university hospital radiology unit.

The indications for performing CT scans were established through a comprehensive clinical assessment. The clinical indications were consistent with established guidelines [12], [13] and determined in an interdisciplinary conference involving board-certified radiologists and cardiologists.

Clinical indications were as follows: a) exclusion of coronary artery disease, b) pre-interventional planning for TAVI evaluation, c) visualization of bypass grafts, d) incidental findings during cardiac catheterization or e) visualization of congenital heart defects. All examinations included a dedicated CT angiography of the coronary arteries. Patients with the above-mentioned clinical indications for CCTA were asked to voluntarily complete a dedicated, standardized PRO questionnaire after their CT examination.

CT examinations were performed on a Dual-Source CT (Somatom Force, Siemens Healthineers, Erlangen, Germany) or a dedicated first-generation clinical Photon-Counting CT (Naeotom Alpha, Siemens Healthineers, Erlangen, Germany).

### 2.2 Patient preparation / questionnaire administration

Patients were initially provided with detailed information about CT examinations, the respective risks and potential side effects and were asked to provide written informed consent. Following the CT examination, they were invited to participate in the voluntary anonymous PRO survey. Following verbal consent, questionnaires were administered using a digital mobile media pad solution (MatePad T10, Huawei, Shenzhen, China) via a dedicated, data-privacy-compliant online survey platform (s2survey.net [SoSci Survey GmbH, Munich, Germany], https://www.soscisurvey.de/en/index).

Special precautions were taken to ensure that questionnaire completion was voluntary, anonymous, and uninfluenced. Participation had no impact on clinical care. Tablets were handed out to patients by study assistants. Final consent or refusal to participate in the survey was recorded digitally and anonymously upon distribution of the survey tablet; at this point, patients were still free to decline participation despite having initially agreed verbally. Participation status and survey responses were not visible to the study assistant collecting the tablet. The questionnaire was completed in a quiet, dedicated CT consultation room.

### 2.3 Questionnaire design and measurement properties

**Fig. 1:**
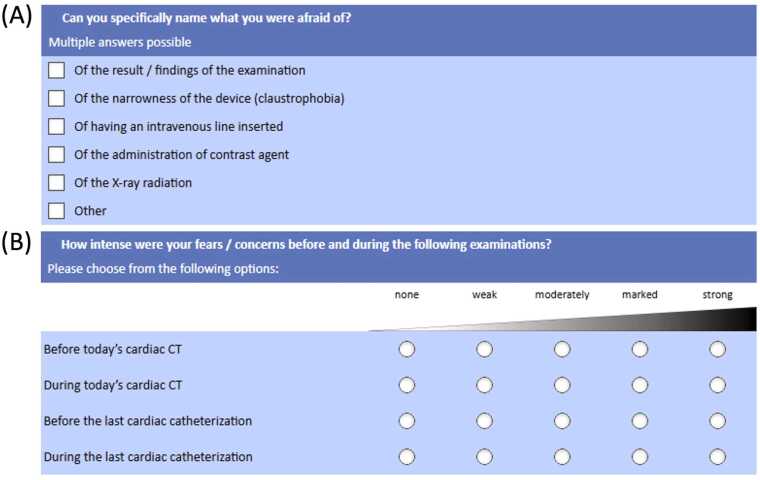
Questionnaire design. Questionnaire design showing (A) questions on patients’ fears related to coronary CT angiography (CCTA), allowing multiple selections, and (B) questions using a Likert scale and visual analogue scale, including comparisons with invasive coronary angiography (ICA).

The survey comprised up to 27 items depending on the patient’s medical history. The questionnaire was locally developed for this study and was not adapted from a previously validated PROM instrument specific to cardiac imaging. It was developed in collaboration with the Institute of Medical Psychology and Medical Sociology at our institution. Item content was informed by general PRO measurement principles (e.g., assessment of anxiety/fear, procedural comfort, overall satisfaction, perceived diagnostic reliability, timeliness of results, and perceived value of physician consultation). Items underwent expert review by medical psychologists and sociologists to ensure relevance and clarity, and were piloted in a small subset of patients to confirm comprehensibility and acceptability. No formal psychometric validation (e.g., factor analysis) and no internal consistency testing (e.g., Cronbach’s alpha) were performed. Accordingly, the PRO results should be interpreted as exploratory and hypothesis-generating. The questions were organized into three thematic categories: patient demographics and medical history, emotional response and perception of the CT examination, and comparison with other medical conditions previously experienced. Responses were collected via predefined options (single- or multiple-selection) or Likert-scale ratings, as illustrated in Fig. 1.

### 2.4 Questionnaire: demographics & examination indications

At the beginning of the survey, patients were asked to provide background information, including age, sex, height, weight, and the indication for their examination. Age, weight, and height were reported within ranges of 5 years, 5 kg, and 5 centimeters, respectively. Regarding the indication for the examination, patients could select from the following options: “to rule out coronary artery disease (CAD),” “for planning a valve implantation (TAVI),” “for visualization of bypass grafts,” “due to an incidental finding during cardiac catheterization,” or “for visualization of a congenital heart defect.”

### 2.5 Questionnaire: evaluation of CCTA and previous diagnostic procedures

To evaluate patient acceptance of CCTA in comparison with ICA and other diagnostic procedures commonly performed in cardiology, patients were first asked to rate the comfort of CCTA and ICA. In addition, several other diagnostic modalities—including transthoracic echocardiography (TTE), transesophageal echocardiography (TEE), cardiac magnetic resonance imaging (CMR), myocardial scintigraphy, CT examinations of other organs, stress electrocardiography (ECG), and blood sampling—were assessed relative to the current CCTA. Patient perceptions of all procedures were evaluated using a Likert scale measuring perceived pleasantness, with response options of 1) very unpleasant, 2) unpleasant, 3) neutral, 4) pleasant and 5) very pleasant. For all procedures except CCTA, an additional option, ‘I have never experienced this procedure,’ was available.

For the direct comparison between CCTA and ICA, patients rated their level of fear before and during each procedure using a Likert scale with the categories 1) none, 2) mild, 3) moderate, 4) marked, and 5) severe. To assess emotional responses after the examinations, patients were asked to select from the options 1) relieved, 2) calm, 3) neutral, 4) worried and 5) frightened.

To further explore patient concerns regarding CCTA, patients were asked whether they had any apprehensions prior to the examination and, if so, to specify their nature. Patients could select from the following options: 1) claustrophobia, 2) contrast agent administration, 3) diagnostic outcome, 4) intravenous catheter, 5) exposure to X-rays, and 6) other. Multiple selections were allowed, and if 6) ‘other’ was chosen, patients were asked to provide a free-text description.

Additionally, patients were asked whether they experienced discomfort during CCTA. Patients could select from 1) application of a venous catheter (4.0%; 6/151), 2) lying uncomfortably on the CT-table (3.3%; 5/151), 3) Multiple selections were allowed, and if 5) ‘other’ was chosen, patients were asked to provide a free-text description.

### 2.6 Questionnaire: which attributes are most important to you when considering coronary CT?

To determine which characteristics of CCTA were most important to patients, patients rated the importance of the following attributes: 1) short examination time, 2) no hospital stay required, 3) rapid availability of results, 4) high diagnostic reliability, 5) non-invasive procedure, and 6) personal consultation following the examination. Ratings were recorded on a five-point Likert scale ranging from “not important” to “very important.”

The overall evaluation of the CCTA examination was obtained using a five-point Likert scale: 5) very good, 4) good, 3), satisfactory, 2) sufficient and 1) insufficient/fail. Each Likert scale was accompanied by a visual analogue scale. To minimize bias from response order effects, the sequence of answer options—ascending or descending in positivity—was randomized across questions.

### 2.7 Outcomes

Given the exploratory nature of this prospective PRO study, no single endpoint was formally pre-specified as primary. However, the manuscript is organized around two main outcome domains: [1] patient-reported fear before and during CCTA (and comparison with prior ICA where available), and [2] patient-reported comfort/pleasantness of CCTA (and comparison with prior ICA where available). All additional analyses (comparisons with other diagnostic procedures, perceived importance of CCTA attributes, and overall ratings) are reported as secondary/exploratory outcomes.

### 2.8 Adverse events

Severe adverse events were defined as events requiring urgent medical intervention or monitoring (e.g., severe contrast reaction/anaphylaxis, hemodynamic instability, respiratory compromise, or other complications necessitating escalation of care). Adverse events were ascertained by patient self-report in the questionnaire. Documentation and observations made by the supervising radiology team during and immediately following the examination could not be correlated with the self-reported events due to the anonymity of the survey.

### 2.9 Inclusion and exclusion criteria

All complete questionnaires were included in the study if they included responses to the identified key indicators. These key indicators encompassed the following queries:

1) General acceptance to participate in the study
2) Fears and concerns associated with the examination
3) Level of the perceived comfort/discomfort during the CCTA
4) Overall rating for CCTA

Furthermore, only questionnaires completed to the final page were incorporated into the analysis. Any questionnaires that did not meet these criteria were systematically excluded from consideration.

### 2.10 Data coding and descriptive statistics

Responses to Likert-scale items were converted to ordinal numerical variables, ranging from 1 (most negative) to 5 (most positive). An exception was made for assessing fear severity before and during CCTA and ICA, using a scale ranging from 1 (none) to 5 (strong). Ordinal outcomes were summarized as absolute frequencies per response category and as mean Likert scores for descriptive purposes. Categorical variables were expressed as absolute numbers and percentages. Likert-scale responses were treated as ordinal, and inferential analyses were therefore performed using non-parametric tests.

### 2.11 Significance statistics

Ordinal data from the Likert-scale assessment of the perceived importance of CCTA characteristics were compared using Friedman’s test. Post hoc pairwise comparisons were conducted using Dunn’s test with a correction for multiple comparisons.

The Wilcoxon signed-rank test was used to compare fear levels before and after CCTA, and before and after ICA. Differences in fear levels prior to CCTA versus ICA, as well as post-procedural feelings, were assessed between procedures using the Mann-Whitney *U* test in the overall sample and the Wilcoxon signed-rank test within the subgroup of patients who underwent both procedures.

P-values < 0.05 were considered statistically significant. All statistical analyses were performed using GraphPad Prism, version 24.0 for Windows (GraphPad Software, Boston, MA, USA).

### 2.12 Handling of missing data

Only questionnaires completed to the final page and containing responses to the predefined key indicators were included. For analyses of specific items, responses were analyzed pairwise/case-wise using available data; no imputation of missing values was performed.

The study was IRB–approved (reference number 286/19) and was conducted in accordance with the Declaration of Helsinki. All patients provided written informed consent.

## 3 Results

### 3.1 Study cohort

Of the 153 patients who underwent CT and agreed to participate in the survey, two did not complete the questionnaire and were therefore excluded. A total of 151 questionnaires were included in the final analysis.

**Fig. 2:**
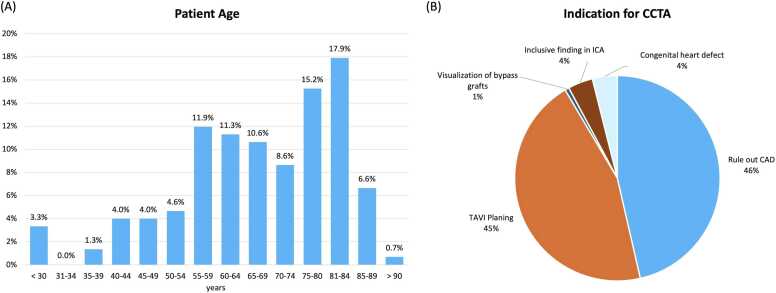
Patient age and indication for CCTA. (A) Patient age with a bar chart of patient counts per age group, and (B) a pie chart showing clinical indications for CCTA, mainly to rule out coronary artery disease (CAD) or plan transcatheter aortic valve implantation (TAVI). ICA: invasive coronary angiography.

The highest proportion of patients were aged 81–85 years (17.9%). The most common weight range was 80–84 kg (16.6%) and the most frequent height range was 170–174 cm (25.2%). See Fig. 2. Detailed demographic data are provided in Table 1.

**Table 1:** Patients’ demographic data.

**Height**	No. of patients (%)
< 155 cm	4 (2.6%)
155–159 cm	11 (7.3%)
160–164 cm	18 (11.9%)
165–169 cm	21 (13.9%)
170–174 cm	38 (25.2%)
175–179 cm	25 (16.6%)
180–184 cm	20 (13.2%)
185–190 cm	12 (7.9%)
> 190 cm	2 (1.3%)

**Weight**	No. of patients (%)
< 45 kg	1 (0.7%)
45–49 kg	1 (0.7%)
50–54 kg	3 (2.0%)
55–59 kg	5 (3.3%)
60–64 kg	17 (11.3%)
65–69 kg	17 (11.3%)
70–74 kg	18 (11.9%)
75–79 kg	12 (7.9%)
80–84 kg	24 (15.9%)
85–89 kg	17 (11.3%)
90–94 kg	5 (3.3%)
95–99 kg	8 (5.3%)
100–104 kg	8 (5.3%)
105–109 kg	7 (4.6%)
> 110 kg	7 (4.6%)

The patients’ demographic data, including the number and percentage across height and weight ranges, largely follow a normal distribution.

Previous CT imaging experience was reported by 83 of 151 patients (55%) for CT in general and by 14 of 151 patients (9.3%) for prior coronary CT angiography (CCTA). Invasive coronary angiography (ICA) had been performed in 69 of 151 patients (45.7%) before the current CCTA. A total of 45 patients (65.2%) underwent ICA within one year before CCTA. Additionally, one patient (1.4%) underwent ICA 1–2 years earlier, six patients (8.7%) 3–4 years prior, four patients (5.8%) 5–10 years earlier, and thirteen patients (18.8%) more than ten years earlier.

The most common indications for CCTA were suspected coronary artery disease (CAD) in 71 of 151 (47%) patients and preprocedural evaluation for transcatheter aortic valve implantation (TAVI) in 69 of 151 (45.7%) patients (Fig. 2).

### 3.2 Concerns regarding CCTA

The majority of patients (83.4%; 126/151) reported no concerns prior to undergoing CCTA.

Among those who did have concerns, 12 of 151 patients (7.9%) were worried about the confined space of the CT scanner, 11 (7.3%) about potential diagnostic findings, 6 (4.0%) about the administration of contrast media, and 1 (0.7%) about radiation exposure. Nine patients (6.0%) reported other, unspecified concerns. Notably, none of the patients expressed concern regarding the placement of the intravenous catheter

### 3.3 Feelings prior to and during CCTA and ICA

#### 3.1 Fear prior to the examination

General feelings of fear *prior to* the respective examination were reported by 39.7% (60/151) of CCTA vs. 66.7% (46/69) of ICA patients, p < 0.001.

Weak or moderate fear *prior to* the respective examination was reported by 33.1% (50/151) of CCTA vs. 40.6% (28/69) of ICA patients.

Marked or strong fear *prior to* the examination was reported by 6.6% (10/151) of CCTA vs. 26% (18/69) of ICA patients.

Fear prior to CCTA (overall mean: 1.68 / subgroup CT and ICA: 1.54) was significantly lower than fear prior to ICA (mean: 2.43) in both groups, in those who solely underwent CCTA compared to ICA-group and in those who underwent both, CCTA and ICA, p < 0.001, respectively.

#### 3.2 Fear during the examination

General feelings of fear *during the* respective examination were reported by 27.8% (42/151) of CCTA vs. 63.8% (44/69) of ICA patients, p < 0.001.

Weak or moderate fear *during* the respective examination was reported by 25.8% (39/151) of CCTA patients vs. 37.7% (26/69) of ICA patients.

Marked or strong fear *during* the examination was reported by 2% (3/151) of CCTA patients vs. 26% (18/69) of ICA patients.

**Fig. 3:**
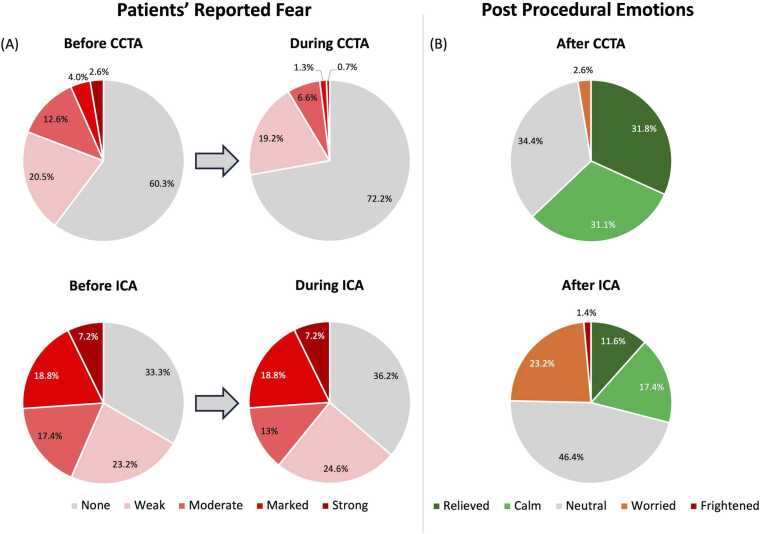
Emotions in the process of CCTA and ICA. (A) Patients’ fear before and during coronary CT angiography (CCTA) and invasive coronary angiography (ICA) is shown as percentages in a pie chart. Fear is less pronounced among patients undergoing CCTA than among those undergoing ICA. In patients undergoing CCTA, fear decreases during the examination, whereas it remains largely unchanged in patients undergoing ICA. (B) Patients’ emotions following CCTA and ICA are also shown, mainly displaying positive feelings like relief or calmness after CCTA, with almost no negative emotions reported. In contrast, patients generally report neutral feelings after ICA, with positive and negative emotions distributed nearly equally.

Overall fears reported prior to and during the examination decreased in CCTA patients from 1.68 to 1.39 (p < 0.001), while they remained essentially unchanged from 2.43 to 2.36 in ICA patients (p = 0.528). The reported fears are displayed in detail in Fig. 3.

After undergoing the respective examinations, relief was reported by 31.8% (48/151) of CCTA vs. 11.6% (8/69) of ICA patients. Calmness was reported by 31.1% (47/151) of CCTA vs. 17.4% (12/69) of ICA patients. Neutrality was reported by 34.4% (52/69) of CCTA vs. 46.4% (32/69) of ICA patients. Feelings of worries were reported by 2.6% (4/151) of CCTA vs. 23.2% (16/69) of ICA patients. State of fright was reported by none (0/151) of CCTA vs. 1.4% (1/69) of ICA patients. The detailed progression of emotions before and during the respective examinations is shown in Fig. 3.

The difference between overall mean rating scores for CCTA (3.92) and ICA (3.14) was statistically significant for all patients, as well as for those who underwent both procedures (CCTA mean score: 3.87) (p < 0.001, respectively).

### 3.4 Comfort during CCTA

Only a minority of patients (13.2%;20/151) experienced uncomfortable situations, whereas the majority (86.8%; 131/151) did not.

Reported inconveniences during CCTA procedure were a) the application of a venous catheter (4.0%; 6/151), b) lying uncomfortably on the CT-table (3.3%; 5/151), c) duration of the examination (1.3%); 2/151) and waiting period prior to the examination (2.6%; 4/151). Six percent (9/151) of patients reported other inconveniences, including pain during contrast application in 1.9% (3/151), nausea in 1.3% (2/151) or ‘scary feeling’ during contrast application in 1.3% (2/151), over-head positioning of the arms in 0.7% (1/151) and pre-procedural fasting in 0.7% (1/151).

### 3.5 Comparison of CCTA to other medical procedures

Most patients perceived the CCTA as pleasant or very pleasant (82%; 124/151) with a mean Likert score of 4.3, while most patients perceived the ICA as unpleasant or very unpleasant (71%; 49/69); with a mean Likert score of 2.0 (p < 0.001).

In comparison to CCTA, TTE was perceived as neutral (mean score: 3.3), TEE as neutral to unpleasant (mean score: 2.5), CMR as neutral (mean score: 2.9), myocardial scintigraphy as mostly neutral (mean score: 2.7), stress-ECG as neutral to unpleasant (mean score: 2.6), CT-scan of another organ as neutral (mean score: 3.0), and getting a blood sample taken as neutral (mean score: 3.1).

In summary, TEE and stress-ECG were generally perceived as more uncomfortable than CCTA (Likert mean: 2.5 and 2.6, respectively).

**Fig. 4:**
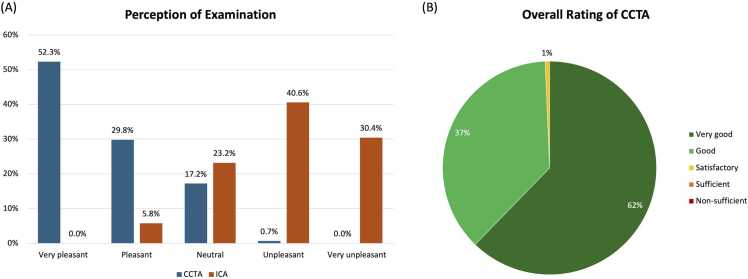
Perception of CCTA and ICA. (A) Perception of coronary CT angiography (CCTA) and invasive coronary angiography (ICA), displayed in absolute rating numbers, is compared via bar chart. Most patients perceived CCTA as mostly (very) pleasant, while ICA is perceived as mostly (very) unpleasant. (B) Overall rating for CCTA shows high acceptance, as nearly all patients (99%) rated it as very good or good.

Detailed results of the inter-procedural comparison are provided in Table 2 and Fig. 4.

**Table 2:** Reception of different diagnostic procedures in comparison to CCTA.

**Procedure**	**Very pleasant**	**Pleasant**	**Neutral**	**Unpleasant**	**Very unpleasant**
**ICA**	0 (0.0%)	4 (5.8%)	16 (23.2%)	28 (40.6%)	21 (30.4%)
**TTE**	10 (9.0%)	21 (18.9%)	70 (63.1%)	7 (6.3%)	3 (2.7%)
**TEE**	2 (5.7%)	5 (14.3%)	10 (28.6%)	9 (25.7%)	9 (25.7%)
**CMR**	4 (13.3%)	4 (13.3%)	11 (36.7%)	8 (26.7%)	3 (10.0%)
**Myocardial scintigraphy**	1 (9.1%)	0 (0.0%)	7 (63.6%)	1 (9.1%)	2 (18.2%)
**CT scan of another organ**	3 (3.6%)	9 (10.8%)	61 (73.5%)	6 (7.2%)	4 (4.8%)
**Stress-ECG**	8 (6.6%)	17 (14.0%)	42 (34.7%)	27 (22.3%)	27 (22.3%)
**Blood sampling**	10 (6.8%)	14 (9.5%)	103 (69.6%)	18 (12.2%)	3 (2.0%)

Displayed is the total number and percentage of ratings of how patients perceived different diagnostic procedures compared to the current coronary CT angiography (CCTA). While most procedures have their rating peak in ‘neutral’ compared to CCTA, including transthoracic echocardiography (TTE), transesophageal echocardiography (TEE), cardiac magnetic resonance imaging (CMR), myocardial scintigraphy, a CT-scan of another organ and blood sampling, invasive coronary angiography shows significantly higher discomfort compared to CCTA. Stress electrocardiography (ECG) is generally perceived as less comfortable than CCTA.

### 3.6 Attributes important to patients

All selectable attributes of CCTA were described as mainly important. The most important characteristic was the high reliability of the examination, chosen as ´very important´ or ´important´ by 93.4% of the patients (very important: 86.8%). Nevertheless, all characteristics were chosen to be very important or important by > 70% of all patients; details are shown in Table 3.

**Table 3:** Important characteristics of CCTA according to patients.

**Characteristics**	**Very important**	**Important**	**Neutral**	**Less important**	**Not important**
**Short examination time**	69 (45.7%)	37 (24.5%)	36 (23.8%)	4 (2.6%)	5 (3.3%)
**No hospital stay necessary**	80 (53.0%)	36 (23.8%)	22 (14.6%)	4 (2.6%)	9 (6.0%)
**Prompt results**	103 (68.2%)	26 (17.2%)	20 (13.2%)	0 (0.0%)	2 (1.3%)
**High reliability of the examination**	131 (86.8%)	10 (6.6%)	9 (6.0%)	0 (0.0%)	1 (0.7%)
**Non-invasive procedure**	94 (62.3%)	30 (19.9%)	25 (16.6%)	1 (0.7%)	1 (0.7%)
**Personal consultation after the examination**	103 (68.2%)	23 (15.2%)	23 (15.2%)	2 (1.3%)	0 (0.0%)

The total number and percentage of ratings showing how important patients find individual characteristics of coronary CT angiography are displayed. All positive characteristics are seen as important, but high reliability, quick result communication, and personal consultation after the exam are rated as most important.

There were significant differences between the characteristics (p < 0.001). In detail, significant differences were observed between the high reliability of the examination and other features, including a short examination time (p < 0.001), no hospital stay necessary (p < 0.001), and a non-invasive procedure (p = 0.009). Further significant differences were found between short examination time and prompt result (p = 0.0032), and between short examination time and personal consultation after examination (p = 0.009).

### 3.7 Overall rating

Nearly all ratings for CCTA were positive, with 62% ‘very good’ ratings, 37% ‘good’ ratings, and only 1% ‘satisfactory’ rating. No patient rated the examination as ‘sufficient’ or ‘non-sufficient/failed’ (Fig. 4). The mean rating score was 4.62.

## 4 Discussion

This study provides important insights into PROs regarding CCTA compared with other diagnostic procedures, particularly ICA. The results demonstrate a strong patient preference for CCTA, consistent with its increasing role in the evaluation of CAD.

A key finding is that, among patients who had previously undergone invasive coronary catheterization, CCTA was rated more favorably than ICA. The non-invasive nature of CCTA likely contributes to greater patient comfort, reduced procedural anxiety, and shorter recovery times, which may explain the strong preference observed in our cohort. In contrast, catheterization carries risks such as vascular complications and procedural discomfort, further accounting for its lower acceptance among patients [14], [15].

Patient concerns regarding CCTA were generally low, with only 39.7% of patients reporting apprehensions beforehand CT, compared to 66.7% prior ICA, suggesting that CCTA is well tolerated and considered a low-risk procedure. Previous studies have indicated that patients experience anxiety prior to CCTA [5] and that concerns about radiation exposure and contrast-related adverse effects are common barriers to its acceptance [16], [17], [18]; however, our findings suggest that these issues may be less impactful in real-world clinical practice. Conversely, a notable proportion of patients reported fears related to the narrowness of the scanner (7.9%) and the potential diagnostic findings (7.3%). This emphasizes an important psychological aspect of diagnostic testing: while patients may not be overly concerned about the procedural risks themselves, they often experience anxiety regarding uncomfortable situations, possible results, and their implications for health outcomes. These results underscore the importance of effective pre-imaging patient communication to address uncertainties and alleviate anxiety [19], [20].

The most valued aspects of CCTA identified in our study were the reliability of diagnostic results, the possibility of a prompt result, and post-scan consultation with the radiologist, all of which were rated as very important by the vast majority of patients. This emphasizes the importance of accurate and conclusive diagnostic information for patient satisfaction. Reliable results are crucial for guiding subsequent clinical decision-making, and patients’ recognition of this underscores their significant trust in CCTA's diagnostic capabilities. Furthermore, the high value placed on the radiologist consultation underlines the necessity for patient-centered communication. Earlier studies have shown that patient comprehension, compliance, and satisfaction improve when imaging results are communicated and explained by healthcare professionals after the examination [21], [22], [23], [24]. This indicates that incorporating structured post-examination discussions into routine clinical practice could enhance the patient experience and trust in CCTA.

Although a relevant number of patients (39.7%) perceived fear immediately before the CCTA, most (33.1%) described this fear as weak or moderate. Additionally, the perceived fear among patients undergoing CCTA decreased significantly during the examination (p < 0.001), indicating that pre-procedural apprehension may be greater than what is ultimately experienced during the scan. This finding strengthens the assumption that patients who have undergone CCTA are likely to be compliant with follow-up examinations. In contrast, 66.7% of patients reported fear immediately before ICA, with a substantial number (26%) reporting marked or strong fear. Moreover, the level of fear in these patients did not significantly decrease during the examination (p = 0.528); therefore, the experience during the procedure appears to meet the patients’ negative expectations. Consistent with these findings, in our study, patients reported greater satisfaction after CCTA than after ICA. Accordingly, most patients afterwards described the experience as (very) pleasant (82%). In contrast, ICA was experienced as mostly (very) unpleasant (71%), indicating higher patient-reported comfort and acceptance for CCTA in this cohort.

Compared to CCTA, most patients perceive other non-invasive diagnostic procedures as neutral. Considering its non-invasive nature and generally positive patient experience, comparable to other standard non-invasive diagnostic methods, CCTA fits well into the diagnostic algorithm for patients with suspected CAD. The low satisfaction score for ICA highlights an essential challenge in contemporary cardiology. While ICA remains the gold standard for invasive coronary assessment, especially when interventional revascularization is needed, its discomfort, invasiveness, and associated risks may discourage patient acceptance, particularly when CAD is an exclusion diagnosis with intermediate pretest probability and only non-severe clinical symptoms are present.

Our results are consistent with the existing literature, which has shown that CCTA provides greater patient safety and diagnostic utility than ICA [25], [26], [27]. CCTA offers high diagnostic accuracy for ruling out obstructive CAD, CAD follow-up, planning TAVI and bypass grafts, thereby minimizing the need for unnecessary invasive procedures [28], [29]. Moreover, studies have emphasized the role of CCTA in optimizing clinical decision-making and cost-effectiveness, especially in patients with low-to-intermediate pretest probability of CAD [29], [30], [31].

Not only can CCTA, due to its high diagnostic sensitivity, spare many patients invasive cardiac catheterization and its potential complications, but its non-invasive nature, short procedural duration, wide availability, and high patient acceptance also offer the possibility of providing broader access to CAD diagnostics and improving compliance in both diagnostics and therapy.

Our findings further underscore the significance of incorporating patient-reported outcomes into the assessment of imaging modalities, strengthening the case for the broader clinical adoption of CCTA.

Our study has limitations. First, the questionnaire was administered only after completion of the CCTA examination. Therefore, emotions and fears reported for the period “before” and “during” CCTA represent retrospective assessments made immediately after the scan. In addition, comparisons with ICA and other procedures rely on patient recollection of experiences that may have occurred months or years earlier. This design introduces potential recall bias, contrast bias, and temporal-distance effects, which may particularly affect comparisons of subtle emotional states such as fear and discomfort. Consequently, statements regarding changes in fear from “before” to “during” CCTA and comparisons between CCTA and ICA should be interpreted as patient-reported perceptions rather than as causal effects of either procedure. Secondly, only the questionnaires of 151 patients who participated in this study fulfilled the inclusion criteria. This number may not be sufficient to generalize the results. The two patients who did not complete the survey may have been dissatisfied with the examinations or had other reasons for not participating, which cannot be addressed in this study. Third, this study was conducted at a single center with two CT scanners for CCTA. In the future, multicenter studies with a larger patient cohort are necessary to confirm the current results. The fourth limitation is that the data originate from a single survey and represent only a snapshot of patients’ experience with CCTA. To account for the increasing integration of CCTA into everyday clinical practice for CAD diagnosis, longitudinal studies that include long-term outcomes and possible follow-up examinations are required. Finally, our cohort reflects a tertiary-care population with a relatively high median age and a substantial proportion of examinations performed for TAVI planning. These characteristics may influence baseline anxiety, perceived procedural risk, and willingness to undergo diagnostic testing. Therefore, the present findings may not fully generalize to younger ambulatory chest pain clinic populations or lower-risk diagnostic settings, in which expectations and perceived burdens may differ.

In conclusion, our study highlights patients’ preference for CCTA over ICA, underscoring its value as a non-invasive, reliable, and well-tolerated diagnostic modality for CAD assessment, which may enhance patient comfort and adherence during both diagnosis and treatment. A post-CT consultation with the physician is considered particularly important by patients and may further enhance the acceptance of the procedure.

## 5 CRediT authorship contribution statement

**Timm Dirrichs:** Conceptualization, Data curation, Funding acquisition, Methodology, Project administration, Supervision, Writing – review & editing. **Robert Siepmann:** Investigation, Methodology, Validation. **Michael Frick:** Conceptualization, Methodology, Supervision, Validation. **Eric Corban:** Data curation, Investigation, Validation. **Christiane Kuhl:** Conceptualization, Funding acquisition, Project administration, Resources, Supervision, Validation. **Marc S. von der Stück:** Conceptualization, Data curation, Formal analysis, Investigation, Methodology, Project administration, Software, Validation, Visualization, Writing – original draft, Writing – review & editing.

## 6 Ethics

Institutional Review Board approval was obtained from the ethical committee of the Medical Faculty of RWTH Aachen University (EK 286/19) on August 22nd, 2019, and was conducted in accordance with the Declaration of Helsinki. All participants provided written informed consent.

## 7 Data sharing statement

Data generated or analyzed during the study are available from the corresponding author upon reasonable request.

## 8 Funding

This research did not receive any specific grant from funding agencies in the public, commercial, or not-for-profit sectors.

## Declaration of Competing Interest

The authors declare that they have no known competing financial interests or personal relationships that could have appeared to influence the work reported in this paper.
